# Self-Administered Gerocognitive Examination: longitudinal cohort testing for the early detection of dementia conversion

**DOI:** 10.1186/s13195-021-00930-4

**Published:** 2021-12-06

**Authors:** Douglas W. Scharre, Shu ing Chang, Haikady N. Nagaraja, Natalie C. Wheeler, Maria Kataki

**Affiliations:** 1 Division of Cognitive Neurology, Department of Neurology, The Ohio State University Wexner Medical Center, 395 W. 12th Ave., 7th Floor, Columbus, OH 43210 USA; 2 Division of Biostatistics, College of Public Health, The Ohio State University, Cunz Hall, Columbus, OH 43210 USA; 3 Present Address: Department of Neurology, University of Wisconsin School of Medicine and Public Health, Madison, WI 53705 USA

**Keywords:** Self-Administered Gerocognitive Examination (SAGE), MMSE, Mild cognitive impairment (MCI), Dementia, Annual rate of change, Dementia conversion, Self-administered cognitive assessment

## Abstract

**Background:**

Significant cognitive changes as individuals’ age are not being identified in a timely manner, delaying diagnosis and treatments. Use of brief, multi-domain, self-administered, objective cognitive assessment tools may remove some barriers in assessing and identifying cognitive changes. We compared longitudinal Self-Administered Gerocognitive Examination (SAGE) test scores to non-self-administered Mini-Mental State Examination (MMSE) scores in 5 different diagnostic subgroups.

**Methods:**

A cohort study evaluating annual rates of change was performed on 665 consecutive patients from Ohio State University Memory Disorders Clinic. Patients with at least two visits 6 months apart evaluated with SAGE and MMSE and classified according to standard clinical criteria as subjective cognitive decline (SCD), mild cognitive impairment (MCI), or Alzheimer’s disease (AD) dementia were included. The pattern of change in SAGE scores was compared to MMSE. One way and repeated measures ANOVA and linear regression models were used.

**Results:**

Four hundred twenty-four individuals (40 SCD, 94 MCI non-converters to dementia, 70 MCI converters to dementia (49 to AD dementia and 21 to non-AD dementia), 220 AD dementia) met inclusion criteria. SAGE and MMSE scores declined respectively at annual rates of 1.91 points/year (*p* < 0.0001) and 1.68 points/year (*p* < 0.0001) for MCI converters to AD dementia, and 1.82 points/year (*p* < 0.0001) and 2.38 points/year (*p* < 0.0001) for AD dementia subjects. SAGE and MMSE scores remained stable for SCD and MCI non-converters. Statistically significant decline from baseline scores in SAGE occurred at least 6 months earlier than MMSE for MCI converters to AD dementia (14.4 vs. 20.4 months), MCI converters to non-AD dementia (14.4 vs. 32.9 months), and AD dementia individuals (8.3 vs. 14.4 months).

**Conclusions:**

SAGE detects MCI conversion to dementia at least 6 months sooner than MMSE. Being self-administered, SAGE also addresses a critical need of removing some barriers in performing cognitive assessments. Limitations of our single-site cohort study include potential referral and sampling biases. Repetitively administering SAGE and identifying stability or decline may provide clinicians with an objective cognitive biomarker impacting evaluation and management choices.

## Background

Cognitive complaints are common in older persons [1]. However, patients present to physicians an average of two to four years after definite cognitive symptoms begin [2–6]. Approximately two-thirds of patients have cognitive scores in dementia ranges when first assessed [5–8], suggesting that less severe cognitive symptoms may have been occurring for years. It is critical for providers to more easily recognize symptoms of brain dysfunction at the mild cognitive impairment (MCI) or early dementia stage [9–11].

MCI can be a prodromal stage of a degenerative dementia or caused by other conditions that may be treatable, modifiable, or reversible [12–14]. Patients diagnosed with MCI do not always progress to dementia. The conversion rate ranges from 21 to 61% in specialist settings followed for at least 3 years [15, 16]. A random-effects meta-analysis including studies from community-based settings demonstrated that the cumulative incidence for the development of dementia in individuals with MCI and those described as cognitively impaired without dementia, older than age 65, and followed for 2 years was 14.9% [17].

Early identification of MCI and dementia is enhanced greatly by brief (10 to 15 min), office-based, multi-domain objective cognitive assessments [18] including the Mini-Mental State Examination (MMSE) [19] and Self-Administered Gerocognitive Examination (SAGE) [20] to detect the degree, type, and changes over time of deficits. SAGE has been shown to be a reliable instrument for detecting cognitive impairment based on gold standard clinical and neuropsychological assessments (ROC AUC of 0.92, 95% specificity, and 79% sensitivity) [20]. SAGE is self-administered and can be taken at a person’s home, in a physician’s office, or virtually anywhere. It requires no special equipment—only pen and paper. The examinee fills out the test in ink without the assistance of others. If any questions are raised by the examinee regarding the test, they are simply told to “Do the best that you can.” Timely detection of cognitive impairment may result in earlier diagnosis and treatment use and lead to increased supervision of the individual. Self-administered tests like SAGE, easily given in any healthcare setting, and sensitive enough to discern those with MCI or early dementia, are few in number [20–25].

We retrospectively compared the utility of longitudinal SAGE test scores from a memory disorders clinic population in an 8-year study to MMSE test scores in 5 different diagnostic subgroups. We also describe the annual rate of change of SAGE and MMSE in different cognitive subgroups (subjective cognitive decline (SCD), MCI, dementia converters of all types, and Alzheimer’s disease (AD) dementia). We hypothesized that the test characteristics of the self-administered SAGE with its more challenging questions and more robust evaluation of executive abilities compared to the MMSE would have less of a ceiling effect and therefore a faster rate of decline in those with very mild cognitive impairments to be able to predict dementia conversion sooner than the MMSE.

## Methods

### Research design and participants

A retrospective chart review was performed on 655 consecutive Memory Disorders Clinic patients seen at The Ohio State University Wexner Medical Center and followed over time up to 8.8 years. Typically, our cognitive follow-up patients are seen every 6 months and receive the SAGE and MMSE at every visit. At each visit, the patient is taken into a separate room by themselves (no informants or family members). A psychometrician administers the MMSE and then hands the 4-page paper SAGE test to the patient with an ink pen. The instructions are simply to answer the questions in ink as best as you can. The individual is left in the room alone and the psychometrician returns in about 15 min to pick up the completed SAGE test and scores it. The cognitive domains tested with the 11-item SAGE include orientation, language, calculations, memory, abstraction, executive, and constructional abilities. The MMSE does not test abstractions nor executive abilities. On average it takes about 7–10 min to administer the MMSE and about 10–15 min for patients to take the SAGE on their own. SAGE has four interchangeable forms designed to reduce learning effects from multiple administrations. A complete description of the SAGE test characteristics is published elsewhere [20]. Patients with sufficient vision and English literacy having at least two visits evaluated with SAGE and MMSE and seen in follow-up (at approximately six-month intervals) were included. Patients with baseline MMSE score less than five or SAGE score less than two (not meaningful for a change over time analysis), age under 50, mental retardation, epilepsy, brain tumor, schizophrenia, ADHD, and non-AD dementia or mixed dementia cases were excluded. The remaining 424 individuals could be divided into categories of SCD, MCI, or AD dementia.

### Diagnostic criteria of dementia, AD, MCI, SCD, and MCI converters

The CERAD neuropsychological battery [26] performed by all patients was the sole basis of determination of normal or impaired cognition for all our baseline SCD and MCI patients. The CERAD neuropsychological battery we used, published by Chandler [26], includes the subtests of verbal fluency, modified Boston Naming Test, word list learning, constructional praxis, word list recall, and word list recognition discriminability, but does not include the MMSE. Activities of daily living (ADL) at each visit were assessed. Specifically, for each patient at every visit, we surveyed the following twenty ADL: driving, finances/bill paying, shopping, cooking, microwave, hobbies or handiwork, lawn or garden work, computer, laundry, other household appliances not specifically itemized, housekeeping, dish washing, cell phone, land telephone, TV remote controller, medication management, dressing, grooming, feeding, and toileting. Standardized options for the driving ADL were as follows: performs independent, drives only locally, able to perform with difficulty, unable to perform, no longer performs, and never did. Standardized options for all other ADL were as follows: performs independently, requires promoting, able to perform with difficulty, requires mild assistance, requires moderate assistance, unable to perform, no longer performs, and never did. Any decline or impairment was considered significant if it was based on loss of function due to cognitive abilities and not if caused by physical conditions (e.g., arthritis or injury). MCI converters were those classified as MCI who later in time met standard criteria for dementia. Fellowship-trained dementia specialists diagnosed all patients as AD dementia, non-AD dementia, MCI, or SCD based on standard clinical criteria as outlined below. Regularly scheduled clinical consensus conferences are held with all the cognitive neurologists to harmonize the use of the clinical criteria and methods for diagnosis at our center.

All baseline AD dementia patients (non-converters) and all baseline MCI patients who converted to AD dementia in this study met or eventually met, respectively, the clinical diagnosis of probable AD according to the National Institute of Neurologic and Communicative Disorders and Stroke/Alzheimer’s Disease and Related Disorders Association [27] and DSM-IV TR [28] criteria. Baseline MCI patients who converted to non-AD dementia or multiple etiology-related dementia were defined as those meeting the standard clinical diagnosis of dementia according to DSM-IV TR criteria [28] and not meeting the criteria above for probable AD. All dementia individuals must have had significant impairment in functional abilities including the need for hands-on assistance in some ADL by their last visit.

Individuals with impaired cognition based on CERAD testing and normal or slight impairment in functional abilities not requiring any hands-on assistance in ADL by their last visit were classified as MCI. This definition of MCI is consistent with standard clinical criteria [29].

Individuals with normal cognition based on CERAD testing and having normal functional abilities based on the ADL scale at all their visits with only subjective cognitive complaints were classified as SCD [30].

### Statistical analyses

**Fig. 1:**
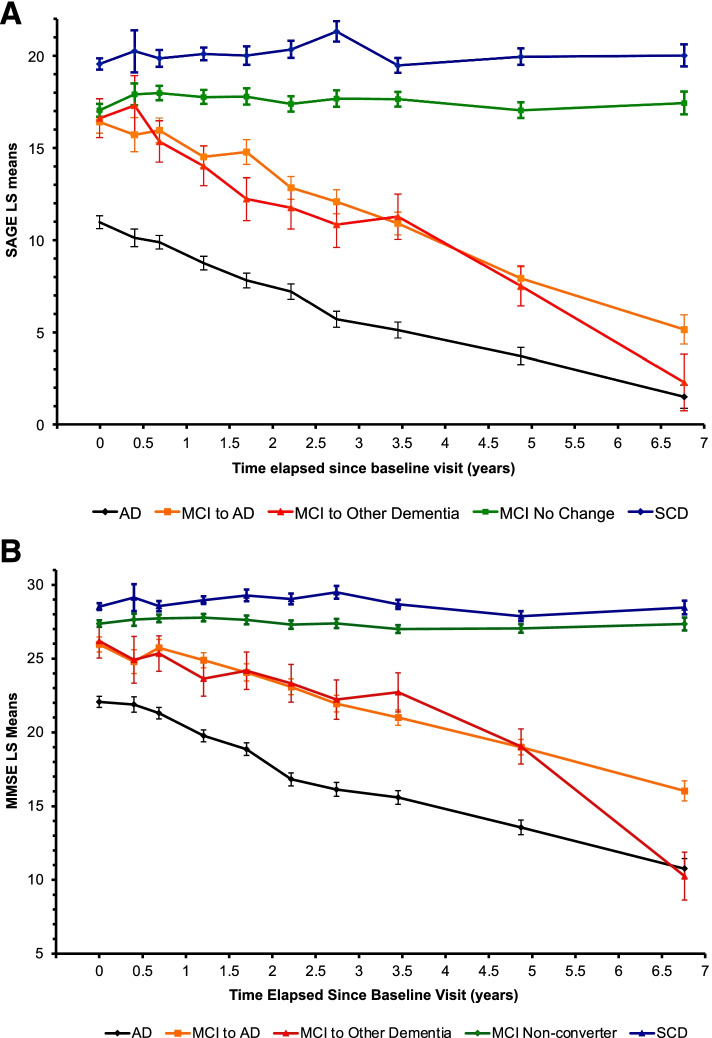
**A** SAGE score changes over time. SAGE test least squares mean scores with standard error bars for each diagnostic group. Abbreviations: AD, Alzheimer’s disease dementia; MCI, mild cognitive impairment; SAGE, Self-Administered Gerocognitive Examination; SCD, subjective cognitive decline. **B** MMSE score changes over time. MMSE test least squares mean scores with standard error bars for each diagnostic group. Abbreviations: AD, Alzheimer’s disease dementia; MCI, mild cognitive impairment; MMSE, Mini-Mental State Examination; SCD, subjective cognitive decline

Baseline means were compared using ANOVA models and proportions were compared using chi-square tests (Table 1). Longitudinal changes were examined two ways. Using actual time of measurement as a continuous predictor, the average annual score change (Table 2) was estimated as the slope of the best-fit random slope and intercept linear regression model for each of the subgroups. Slopes were compared using *Z* tests. Next, baseline visit was labeled V0 and visit numbers V1 to V9 were created to represent respectively time periods 0–0.5, 0.5–1.0, 1.0–1.5, 1.5–2.0, 2.0–2.5, 2.5–3.0, 3.0–4.0, 4.0–6.0, and over 6.0 years from V0. Mixed effect linear models were fit to determine the earliest visit number to detect the decrease in SAGE and MMSE scores (Table 3). Least-squares estimates and their standard errors of the fixed effects were used in Fig. 1. Examination of residuals resulted in the removal of one outlier in the MMSE data from a single visit from one subject. The level of significance was set at 0.05 and for multiple comparisons, Tukey’s HSD test or appropriate Bonferroni correction was applied. JMP 13.0 software (SAS Institute, Cary, NC) was used for the analyses.

## Results

### Participant clinical characteristics

Forty SCD, 94 MCI non-converters, 70 MCI converters, and 220 AD dementia patients met the inclusion and exclusion criteria. Of the 70 patients who converted from MCI to dementia, 49 developed AD dementia and 21 were diagnosed with other non-AD causes of dementia. The other dementia diagnoses were Lewy body dementia (five), vascular dementia (three), normal pressure hydrocephalus (two), vasculitis (two), mixed small vessel vascular dementia and chronic kidney disease (two), frontotemporal dementia (one), cerebral amyloid angiopathy (one), mixed AD and vascular dementia (one), mixed AD and radiation leukoencephalopathy (one), multiple sclerosis (one), uremia (one), and chronic hepatic encephalopathy from hepatitis C (one). Since only two SCD patients converted to MCI over the course of our study, no conclusions regarding this group could be made. Table 1 provides the demographics of our study population. AD dementia and MCI converters were significantly older than our MCI non-converters and SCD patients. Duration of follow-up will naturally be longer for MCI converters than other groups as time is required for conversion. There were no significant differences in the mean duration of follow-up between those patients with AD dementia, MCI non-converters, and SCD (Tukey HSD test for multiple comparisons *p* > 0.05). Our AD dementia patients showed numerically lower educational attainment when compared to MCI converters (Tukey HSD test *p* = 0.0061). Proportion of females among AD dementia patients was not significantly different from MCI converters (*p* ≥ 0.0167). However, there were significantly more female AD patients than MCI non-converters and SCD patients (*p* < 0.0167). Baseline mean SAGE score for SCD was significantly different than MCI non-converters, MCI converters, and AD dementia groups (all *p* < 0.003). However, baseline mean MMSE score for SCD was not significantly different than MCI non-converters (*p* = 0.19) but was significantly different than MCI converters (*p* = 0.0002) and AD dementia groups (*p* < 0.0001). AD dementia patients had significantly lower baseline SAGE and MMSE scores than all other groups (Tukey HSD test *p* < 0.05 for each comparison).

**Table 1:** Demographics

	AD dementia (***n*** = 220)	MCI converter (***n*** = 70)	MCI converter		MCI non-converter (***n*** = 94)	SCD (***n*** = 40)
			To AD dementia (***n*** = 49)	To other dementia (***n*** = 21)		
Age (years), mean (SD) and range	76.9 (8.5) 50–91	75.0 (6.7) 55–91	74.8 (6.1) 55–91	75.4 (8.0) 57–89	69.7 (9.8)^a^ 50–89	69.30 (9.2)^a^ 51–85
Follow-up duration (years), mean (SD) and range	2.8 (1.7) 0.5–7.7	4.0 (1.9)^a^ 1.2–8.7	4.1 (1.9) 1.2–8.7	3.8 (2.0) 1.2–7.0	3.2 (1.9) 0.5–7.8	3.46 (2.0) 1.0–7.7
Education (years), mean (SD) and range	14.4 (3.0)^b^ 4–20	15.7 (2.8)^a^ 10–20	15.9 (2.7) 10–20	15.3 (3.1) 12–20	15.3 (2.6) 10–20	15.6 (2.6) 10–20
Female sex, *N* (%)	135 (61)	35 (50)	25 (51)	10 (48)	39 (42)^c^	16 (40)^c^
Caucasian race, *N* (%)	202 (92)	67 (96)	47 (96)	20 (95)	86 (92)	37 (93)
SAGE baseline, mean (SD) and range (maximum score 22)	11.0 (4.4) 2–22	16.5 (3.1)^a^ 8–22	16.4 (3.1) 9–21	16.6 (3.2) 8–22	17.0 (3.3)^a^ 7–22	19.6 (2.0)^a^ 14–22
MMSE baseline, mean (SD) and range (maximum score 30)	22.1 (3.7) 9–30	26.0 (2.4)^a^ 19–30	26.0 (2.3) 19–30	26.2 (2.8) 20–30	27.4 (1.9)^a^ 21–30	28.5 (1.4)^a^ 23–30

*Abbreviations*: *AD* Alzheimer’s disease, *MCI* Mild cognitive impairment, *MMSE* Mini-Mental State Examination, *SAGE* Self-Administered Gerocognitive Examination, *SCD* Subjective cognitive decline, *SD* Standard deviation

^a^Mean significantly different from the AD mean, from Tukey HSD test in a comparison of AD, MCI converter, MCI non-converter, and SCD groups

^b^Eight missing

^c^Significantly different from the AD proportion from Pearson chi-square test with Bonferroni correction for three comparisons

### Score changes over time

Table 2 outlines the average yearly score changes for SAGE (22 maximum) and MMSE (30 maximum) for the different diagnostic groups. Age and education as potential predictors of either MMSE or SAGE score change over time did not impact the slope estimates. Both SAGE and MMSE scores respectively declined significantly over time at annual rates of 1.91 (*p* < 0.0001) and 1.68 (*p* < 0.0001) for MCI converters to AD dementia, and at annual rates of 2.33 (*p* < 0.0001) and 1.83 (*p* < 0.0001) for MCI converters to other dementia. There was no statistical difference in the rates of decline for the two convertor groups using SAGE (*p* = 0.21) or MMSE (*p* = 0.63). Both SAGE and MMSE scores respectively declined significantly over time at annual rates of 1.82 (*p* < 0.0001) and 2.38 (*p* < 0.0001) for AD dementia patients. Both SAGE and MMSE scores remained similar over time for SCD and MCI non-converters (*p* > 0.05).

**Table 2:** Least squares estimates of the slope for SAGE and MMSE score changes

Subgroup	Change per year (SE)		***P*** value for zero slope	
	SAGE (22 points maximum)	MMSE (30 points maximum)	SAGE	MMSE
MCI to AD dementia	–1.91 (0.14)	–1.68 (0.14)	< .0001	< .0001
MCI to other dementia	–2.33 (0.31)	–1.83 (0.26)^a^	< .0001	< .0001
AD dementia	–1.82 (0.11)	–2.38 (0.14)	< .0001	< .0001
AD dementia, SAGE ≥5 (*n* = 202)	–1.9 (0.11)	–2.4(0.14)	< .0001	< .0001
AD dementia, SAGE 2–4 (*n* = 18)	0.04(0.34)	–2.17(0.68)	0.9161	0.0054
MCI con-converters	0.01 (0.09)	–0.09 (0.05)	0.8907	0.0893
SCD	0.08 (0.08)	–0.01 (0.08)	0.3168	0.8848

*Abbreviations*: *AD* Alzheimer’s disease, *MCI* Mild cognitive impairment, *MMSE* Mini-Mental State Examination, *SAGE* Self-Administered Gerocognitive Examination, *SCD* Subjective cognitive decline, *SE* Standard error

^a^One outlier removed

Figure 1 shows SAGE (Fig. 1A) and MMSE (Fig. 1B) score changes over visits for each of our diagnostic groups.

### Early detection of dementia conversion using SAGE

Table 3 lists the estimates of mean score changes from baseline over time up to nine visits, for AD dementia and Convertor subgroups. Visit number has a significant effect on MMSE and SAGE scores for AD dementia, MCI converter to AD dementia or other dementia groups (*p* < 0.0001), but not for the SCD and MCI non-convertor groups (*p* > 0.05). For SCD and MCI non-convertor groups, the rates of score changes over time were not significant and hence not displayed in Table 3. The baseline visit is designated as V0. Our cognitive follow-up patients are typically evaluated every 6 months. For the first follow-up visit after a new patient visit, if medications were started, patients were usually instructed to come back in 3 months. So, the mean time to the first follow-up visit (V1) was 0.4 years and to the second follow-up visit (V2) was 0.69 years in our cohort. After that, the mean time to subsequent follow-up visits is approximately 0.5 years. After 3 years (V7 to V9), we expanded the visit interval due to the lower number of subject visits (see Table 3).

**Table 3:** Least squares estimates of SAGE/MMSE score mean changes from baseline (V0) (*p* values) for visits V1–V9^a^

Subgroup^**b**^	Test	Visit number (average duration in months since baseline visit V0)								
		V1 (4.8)	V2 (8.3)	V3 (14.4)	V4 (20.4)	V5 (26.6)	V6 (32.9)	V7 (41.4)	V8 (58.4)	V9 (81.1)
**MCI to AD dementia**	SAGE	−0.68 (0.4593)	−0.44 (0.5043)	−1.88 (0.0016)^c^	−1.61 (0.0158)	−3.57^d^	−4.32^d^	−5.51^d^	−8.47^d^	−11.25^d^
	MMSE	−1.17 (0.1544)	−0.24 (0.6862)	−1.07 (0.0440)	−1.89 (0.0015)^c^	−2.87^d^	−4.01^d^	−4.96^d^	−6.96^d^	−9.92^d^
**MCI to other dementia**	SAGE	0.70 (0.6427)	−1.26 (0.1904)	−2.59 (0.0051)^c^	−4.39^d^	−4.85^d^	−5.77^d^	−5.35^d^	−9.10^d^	−14.33^d^
	MMSE^e^	−1.27 (0.3663)	−0.85 (0.3749)	−2.56 (0.0061)	−2.02 (0.0528)	−2.87 (0.0072)	−3.97 (0.0005)^c^	−3.48 (0.0005)	−7.15^d^	−15.94^d^
**AD dementia**	SAGE	−0.85 (0.0406)	−1.09 (0.0001)^c^	−2.22^d^	−3.17^d^	−3.77^d^	−5.27^d^	−5.85^d^	−7.26^d^	−9.46^d^
	MMSE	−0.17 (0.7150)	−0.77 (0.0195)	−2.23^c,d^	−3.29^d^	−5.34^d^	−5.94^d^	−6.48^d^	−8.52^d^	−11.34^d^

*Abbreviations*: *AD* Alzheimer’s disease, *MCI* Mild cognitive impairment, *MMSE* Mini-Mental State Examination, *SAGE* Self-Administered Gerocognitive Examination, *SCD* Subjective cognitive decline

^a^Visit Numbers V1-V9 are labeled as V1: within 6 months; V2: 6–12 months; V3: 12–18 months, V4: 18–24 months, V5: 24–30 months, V6: 30–36 months, V7: 36–48 months, V8: 48–72 months, V9: over 72 months (max 105 months)

^b^None of the mean differences in the MCI (non-converter) and SCD groups was significant

^c^First significant difference; threshold for significance with Bonferroni correction is a *p* value below 0.05/9 = 0.00555

^d^Represents *p* values under 0.0001

^e^One outlier removed

The first significant decline in SAGE scores was observed at visit three (V3) for those who converted to either AD dementia or another dementia (mean of 14.4 months from baseline). The first significant decline in MMSE scores was observed at visit four (V4) for those who converted to AD dementia (mean of 20.4 months from baseline) and at visit six (V6) for those converting to another dementia type (mean of 32.9 months from baseline). Thus, significant changes in SAGE scores for the converter groups occurred at least 6 months earlier than significant changes in MMSE scores.

For those patients who started with AD dementia, the first significant decline in SAGE scores occurred at visit two (V2), a mean of 8.3 months from baseline, whereas the first significant decline for MMSE scores occurs at visit three (V3), a mean of 14.4 months. Again, it was noted that significant changes in SAGE scores occurred over 6 months earlier than significant changes in MMSE scores.

## Discussion

Our findings are based on a study population that is typical of memory disorders clinics at most academic medical centers [31, 32]. Among the 164 patients with baseline MCI, 70 patients converted to dementia, a 43% conversion rate over 3 to 4 years, similar to rates from other academic center-based studies [16, 17]. Our distribution of dementia diagnoses (including 70% AD dementia, 7% Lewy body dementia, and 9% pure or mixed vascular dementia) is comparable to literature reports and incidence studies [16, 28, 33]. Most of the patients did not have PET or CSF markers of AD pathology. However, the longitudinal nature of the study with repeat clinical evaluations increases the likelihood of more accurate eventual diagnostic classifications (AD versus non-AD dementia). Minority individuals were proportionately less represented and higher educated patients were more represented in our sample than the general population, typical of other medical center studies of MCI and dementia [31].

Knowing the average rate of decline of cognitive assessment scores for dementing disorders is very useful for the clinician, patient, and caregivers. Stability status or rate of decline of the cognitive test scores gives the clinician insight into the clinical course of the disorder. Do medications need to be started or adjusted? Are the current treatments helping? Should further evaluations be performed? Are there other factors to consider that may be influencing or impacting the score changes? Is this score change typical for the disease progression? Does the current diagnosis need revisiting? Are clinical trials appropriate at this time? Based on cognitive score changes, clinicians and families may decide it is time to act on safety and supervision needs. This might include, for example, medication oversight, financial assistance, driving limitations, setting up durable Powers of Attorney and other legal arrangements/trusts, change in living arrangements, and enhanced caregiving support.

For SAGE, we see similar, non-significant differences in the annual rates of decline in scores of those MCI individuals that convert to AD dementia (SAGE declining 1.91 points per year) and those with AD dementia (SAGE declining 1.82 points per year). For those AD dementia patients who score over four points on SAGE, the annual rate of decline (1.9 points per year) is nearly identical to the MCI converters to AD dementia group. However, for the 18 AD dementia patients with baseline SAGE under five, MMSE scores still declined significantly over time (*p* = 0.0054), while SAGE scores did not (*p* = 0.92). This suggests a floor effect using SAGE for SAGE scores below five. These are individuals with moderate to severe AD dementia. When individuals are scoring consistently below 5 on their SAGE, this test will not provide useful change over time information.

The annual rate of decline on MMSE for those MCI individuals that converted to AD dementia (MMSE declining 1.68 points per year) is significantly less (not as steep) than for those with AD dementia (MMSE declining 2.38 points per year) (*p* = 0.0006). This observation has been previously reported in the literature [34–36]. This helps to explain why significant SAGE test score declines are detected earlier than MMSE for those individuals that convert from MCI to AD dementia. On the other hand, MMSE is superior to SAGE in detecting cognitive score declines in moderate to severe AD dementia due to SAGE floor effects occurring earlier in the disease course than MMSE floor effects. However, in those individuals with moderate to severe dementia, a decline in ADL abilities is often as good a gauge of dementia progression as a decline in cognitive scores [37].

As seen in Fig. 1, mean SAGE scores of SCD patients were consistently higher than those of MCI patients. Some of these individuals are at risk to develop objective cognitive deficits (MCI and dementia) or may have sub-threshold cognitive impairments that often become apparent only after many years [30, 38–40]. Previous studies have shown that SAGE scores of cognitively normal individuals are significantly different from the SAGE scores of MCI diagnosed individuals [20]. Individuals with MCI should be evaluated for treatable or reversible causes to improve or slow decline of their cognitive issues. It is important, therefore, to have cognitive assessment tools to be able to reliably identify those with MCI.

Figure 1 also shows that the MCI converters to non-AD dementias decline in their SAGE and MMSE scores more unevenly than the MCI converters to AD dementia. While this discrepancy is partly due to the first group having a smaller sample size (*n* = 21 versus *n* = 49), the relative magnitude of the standard error is more than what one expects from the sample size difference. This variability of decline is not surprising as the MCI converters to non-AD dementias represent a variety of distinct dementia conditions ranging from degenerative to vascular to metabolic conditions, most of which have dissimilar and variable clinical courses.

Significant declines in SAGE scores for the MCI converters to both AD and non-AD dementia, and for the AD dementia individuals, occurred at least 6 months earlier than significant changes in MMSE scores (Table 3). Everyone has different cognitive abilities, natural talents, educational achievements, and life experiences. Therefore, it is ideal to have individual baseline assessments prior to any significant decline in cognitive skills for later self-comparison. A significant decline in a person’s cognitive test score over time can be one of the most useful indicators of a new or progressing brain condition. Self-comparison longitudinal assessments also eliminate the educational and cultural biases of the assessment tools for the specific individual.

Practically speaking, for those patients with MCI who will convert to dementia, the first significant decline in mean SAGE scores occurred at a mean of 14.4 months from their baseline visit. From the slope estimates reported in Table 2, we expect an average drop of 2.44 points in their SAGE score in 14.4 months or 2–3 points in 12–18 months. So, if the clinical provider who is regularly obtaining SAGE assessments (suggested to be given when cognitive concerns arise and/or at 6-month intervals) records a 2–3 point drop or more in 12–18 months, this could represent a significant decline in their patient’s score. We carried out an ROC analysis of SAGE score changes (V3–V0) from MCI non-converter and MCI converter groups. With a 2-point decline as a cut-off, SAGE provided a sensitivity of 54% and specificity of 81% for detecting conversion to dementia by the end of the study. Assessment tools that are reliably able to provide an objective measure of clinically meaningful cognitive change over time (essentially a cognitive biomarker) could prompt more timely action by providers in identifying causes and instituting a management plan.

Barriers to providing these initial and repeat assessments mostly revolve around the degree of impracticality of testing and repeat testing in the primary care provider’s clinic setting. Barriers include the length of time to administer the tests, the availability of trained staff to administer the test, the delay in performance of other needed duties important in the efficient running of the clinic by the test administrator who is now unavailable while they are giving the test, the space required to conduct the testing that necessitates separation and noise abatement from other staff and patients, and the need for any specialized testing equipment. Additional barriers include the uncomfortable feeling patients may experience when being “tested” directly in person by someone else, the learning effects from repeat testing impacting score results, and the cost of performing the assessments. These barriers are mitigated by tools like SAGE (free of charge at sagetest.osu.edu, the SAGE test website [41]) allowing for the ease of repeating assessments over time and increasing its practical impact on the early identification of cognitive changes. Administered tests like the MMSE and others are more burdensome in busy clinical settings than the SAGE and consequently are less likely to be administered and repeated regularly over time. Clinical providers wish to provide the best assessments and care to their patients in a timely fashion within their existing time constraints. This study suggests that providers using SAGE instead of the MMSE can expect to identify cognitive changes sooner and be able to achieve that using a self-administered test that utilizes less provider and staff time, does not require any training to administer the test, and can be given outside heavily utilized exam rooms to prevent clinic workflow disruption. SAGE is easily incorporated into clinical provider visits and may result in significantly earlier awareness of new cognitive conditions/concerns leading to new diagnoses, treatment, or management changes. The 11-item SAGE testing the cognitive domains of orientation, language, calculations, memory, abstractions, executive, and constructional abilities has more challenging questions and more robust evaluation of executive abilities compared to the MMSE. This provides SAGE with less of a ceiling effect and a faster rate of decline in those with very mild cognitive impairments. While the SAGE takes on average a bit longer (10–15 min) to complete than the MMSE (7–10 min), self-administered SAGE saves time for any administrator. While this study did not compare SAGE to other multi-domain cognitive assessment tools besides the MMSE, previous research has shown that SAGE scores correlate very well to neuropsychology batteries, the Montreal Cognitive Assessment (MoCA), and to the digital version of SAGE made for tablet use (commercially available at BrainTest.com through a license agreement with The Ohio State University) [42]. For both the digital version of SAGE (called BrainTest®) and the paper assessment, patients perform the test alone without any administrator. From our previous study, the Spearman correlation of paper SAGE versus BrainTest was 0.88 (*p* < 0.0001) [42]. BrainTest and SAGE were related by a line with the slope very close to 1 (*p* = 0.86) suggesting strong evidence that the scaling is identical between the two [42].

There are also barriers to the initial identification of cognitive impairments. Many patients do not seek timely evaluations for their cognitive complaints [2–8]. These delays may be contributed by the individual’s impaired insight, trepidation or embarrassment, or believing they have normal aging. Case-finding, practically given self-administered tests (compared to administered tests) that can be completed before, during, or after a health care provider’s visit may more easily allow the opportunity to identify potential cognitive deficits at an early stage. However, more research with these tools will be required to test that hypothesis.

## Limitations

There are specific limitations related to the SAGE test itself including that assessments of memory abilities in self-administered instruments are challenging, individuals with low vision are unable to fully complete the test, a sixth grade reading proficiency is required, and as no explanations of the test questions are allowed, some individuals may misread/misunderstand a question they would normally get correct. However, such errors could also suggest attentional or executive impairments, which commonly occur in those identified with MCI or dementia. Further studies in low educated cohorts are needed.

There are also limitations inherent to this study. Our research involves a retrospective chart review from an academic Memory Disorders Clinic with referral and sampling biases. More studies need to be done to evaluate non-academic medical center populations, minorities, and those with lower educational attainment. The present study compares SAGE and MMSE given in a clinic setting and this study’s conclusions cannot be extended to SAGE being given remotely compared to the MMSE. However, the self-administered nature of SAGE has the potential to afford a lower barrier to cognitive assessments. The longitudinal nature of the study with repeat self-comparison evaluations would increase the likelihood of a more accurate eventual diagnostic classification. This is a strength of our study. In addition, for our analysis of early detection of dementia conversion, MMSE and SAGE are being compared in the same subject regardless of which diagnostic group they were a part of.

Due to small numbers, we cannot make any conclusions regarding score changes for non-AD dementia conditions and longer longitudinal studies are required to help determine if SAGE could help identify conversion from normal or SCD individuals to MCI conditions.

## Conclusions

In summary, our longitudinal retrospective study revealed that for the MCI converters to dementia and for the AD dementia individuals, significant changes in SAGE scores occurred at least 6 months earlier than significant changes in MMSE scores. The annual rate of decline in SAGE scores, just under two points per year, is similar for individuals who have MCI due to AD and for those with mild to moderate AD dementia while the MMSE declines more slowly for those with MCI due to AD. SAGE aids in the identification of MCI status and is sensitive to cognitive changes over time. A 2–3-point drop or more in SAGE scores in 12–18 months may be significant and should trigger the provider to consider additional diagnoses, evaluations, or management changes. For the clinical provider who longitudinally manages those with cognitive issues, use of SAGE provides a cognitive assessment tool that identifies cognitive changes sooner than MMSE, has advantages of time efficiencies in busy clinical practices, and consequently may more likely be administered and repeated regularly over time. The ease of repetitively giving the self-administered SAGE and identification of score stability or decline may provide clinicians with an objective cognitive biomarker impacting their evaluation and management choices. SAGE has the advantage of self-administration, brevity, four interchangeable forms, and widespread availability to be a factor in overcoming the many obstacles in identifying cognitive changes in patients.

## Data Availability

The datasets used and analyzed during the current study are available from the corresponding author on reasonable request.

## Abbreviations

AD: Alzheimer’s Disease
ADL: Activities of daily living
MCI: Mild cognitive impairment
MMSE: Mini-Mental State Examination
MoCA: Montreal Cognitive Assessment
SAGE: Self-Administered Gerocognitive Examination
SCD: Subjective cognitive decline
SD: Standard deviation
