# Correction to: Self-Administered Gerocognitive Examination: longitudinal cohort testing for the early detection of dementia conversion

**DOI:** 10.1186/s13195-022-00965-1

**Published:** 2022-02-05

**Authors:** Douglas W. Scharre, Shu ing Chang, Haikady N. Nagaraja, Natalie C. Wheeler, Maria Kataki

**Affiliations:** 1 Division of Cognitive Neurology, Department of Neurology, The Ohio State University Wexner Medical Center, 395 W. 12th Ave., 7th Floor, Columbus, OH 43210 USA; 2 Division of Biostatistics, College of Public Health, The Ohio State University, Cunz Hall, Columbus, OH 43210 USA; 3 Present Address: Department of Neurology, University of Wisconsin School of Medicine and Public Health, Madison, WI 53705 USA

**Correction to: Alz Res Ther 13, 192 (2021)**

**https://doi.org/10.1186/s13195-021-00930-4**

Following the publication of the original article [1] the authors requested to add the statement “Douglas W. Scharre is a member of the Scientific Advisory Board of BrainTest Inc. SEZC.” to Dr. Scharre's disclosures under Funding section.

The original article [1] has been updated.
